# Investigation into inconsistent lateralisation of language functions as a potential risk factor for language impairment

**DOI:** 10.1111/ejn.14623

**Published:** 2019-12-14

**Authors:** Abigail R. Bradshaw, Zoe V. J. Woodhead, Paul A. Thompson, Dorothy V. M. Bishop

**Affiliations:** ^1^ Department of Experimental Psychology Radcliffe Observatory Quarter University of Oxford Oxford UK

**Keywords:** developmental disorder, functional transcranial Doppler sonography, hemispheric specialisation, language, lateralisation

## Abstract

Disruption to language lateralisation has been proposed as a cause of developmental language impairments. In this study, we tested the idea that *consistency* of lateralisation across different language functions is associated with language ability. A large sample of adults with variable language abilities (*N* = 67 with a developmental disorder affecting language and *N* = 37 controls) were recruited. Lateralisation was measured using functional transcranial Doppler sonography (fTCD) for three language tasks that engage different language subprocesses (phonological decision, semantic decision and sentence generation). The whole sample was divided into those with consistent versus inconsistent lateralisation across the three tasks. Language ability (using a battery of standardised tests) was compared between the consistent and inconsistent groups. The results did not show a significant effect of lateralisation consistency on language skills. However, of the 31 individuals showing inconsistent lateralisation, the vast majority (84%) were in the disorder group with only five controls showing such a pattern, a difference that was higher than would be expected by chance. The developmental disorder group also demonstrated weaker correlations between laterality indices across pairs of tasks. In summary, although the data did not support the hypothesis that inconsistent language lateralisation is a major cause of poor language skills, the results suggested that some subtypes of language disorder are associated with inefficient distribution of language functions between hemispheres. Inconsistent lateralisation could be a causal factor in the aetiology of language disorder or may arise in some cases as the consequence of developmental disorder, possibly reflective of compensatory reorganisation.

## Graphical abstract

We tested the hypothesis that inconsistent lateralisation across language functions is associated with poorer language skills. In a sample of 104 adults with an overrepresentation of language disorders no difference in language skills was found between consistently and inconsistently lateralised groups; however, developmental disorder individuals were more likely to show inconsistent lateralisation across tasks. The results suggest that inefficient distribution of language functions between hemispheres may be involved in some cases of language disorder.

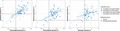

**Abbreviations**
CC‐SR: Communication Checklist—Self‐report
DCD: developmental coordination disorder
DD: development disorder
ERRNI: Expression, Reception and Recall of Narrative Instrument
fMRI: functional magnetic resonance imaging
fTCD: functional transcranial Doppler sonography
LI: laterality index
MCA: middle cerebral artery
NEPSY: a developmental neuropsychological assessment
PD: phonological decision
REDCap: Research Electronic Data Capture
SD: semantic decision
SG: sentence generation
SpLD: specific learning impairment
TOAL‐4: Test of Adolescent and Adult Language
TOWRE: Test of Word Reading Efficiency
vOT: ventral occipitotemporal cortex
WAIS: Wechsler Adult Intelligence Scale
WASI: Wechsler Abbreviated Scale of Intelligence
YAA‐R: York Adult Assessment Battery—Revised

## 1 INTRODUCTION

Language lateralisation refers to the well‐known finding that one hemisphere tends to be more specialised for language processing than the other; typically, this is manifest as greater involvement of the left hemisphere than the right in language. The functional relevance of this lateralisation is as yet unknown, but a long‐standing hypothesis has proposed that a failure to establish lateralisation may be a cause of language impairments (Annett, 1985; Orton, 1937). The approach of most imaging studies in this area has been to compare the incidence of ‘atypical’ laterality in a language impaired group to that in a control group, defined on the basis of whether the expected left‐sided pattern of brain activity is shown during performance of a single lateralising task. Here, we test the novel hypothesis that as well as strength or direction of lateralisation, *consistency* of lateralisation across multiple tasks may have an impact on language development.

### 1.1 Language lateralisation and language ability/impairment

Although there is a strong bias towards left lateralisation of language at the group level, there are considerable individual differences within this (e.g. Mazoyer et al., 2014). ‘Atypical laterality’ is a generic term that is used to describe individuals showing either a right bias or a lack of hemispheric dominance (bilaterality) on standard laterality measurements. Previous research has investigated the association between such individual differences in language lateralisation and language abilities in both participants with typical language development and those with developmental language impairments. In both cases, the results have been mixed.

In participants with typical language development, early imaging work reported no relationship between strength of laterality and language abilities in both adult and child samples (Holland et al., 2001; Knecht et al., 2001; Wood et al., 2004). However, more recent studies have tended to report a positive relationship (Chiarello, Welcome, Halderman, & Leonard, 2009; Everts et al., 2009; Groen, Whitehouse, Badcock, & Bishop, 2012; Mellet et al., 2014). For example, Mellet et al. (2014) reported that weak lateralisation for language (bilaterality) on fMRI was associated with reduced performance on both verbal and non‐verbal tasks relative to individuals showing strong lateralisation, regardless of the direction of that lateralisation. A small number of studies have reported relationships in the opposite direction, which may reflect the nature of the lateralising tasks employed (Bartha‐Doering et al., 2018; van Ettinger‐Veenstra et al., 2010; Hirnstein, Leask, Rose, & Hausmann, 2010).

In participants with language impairment, a number of studies have reported a higher incidence of atypical laterality or lower laterality indices when compared to control groups (Badcock, Bishop, Hardiman, Barry, & Watkins, 2012; Bishop, Holt, Whitehouse, & Groen, 2014; de Guibert et al., 2011; Illingworth & Bishop, 2009; Waldie, Haigh, Badzakova‐Trajkov, Buckley, & Kirk, 2013; Whitehouse & Bishop, 2008). This has suggested the possibility that atypical lateralisation could constitute an endophenotype for language impairment, mediating the relationship between an original etiological factor such as a genetic predisposition and impaired language function (Bishop, 2013). However, a recent large scale study by Wilson and Bishop (2018) failed to replicate an association between reduced laterality and developmental language disorder, suggesting that previous associations in language impaired samples may represent false positives. Overall therefore, the evidence for reduced strength of lateralisation in language impaired samples is equivocal.

### 1.2 Atypical versus inconsistent laterality

Thus far, this line of research has revolved around the distinction between typical left and atypical right/bilateral lateralisation. However, individuals may differ in the organisation of their language systems across the hemispheres in meaningful ways that are not captured by this simple typical–atypical dichotomy. More recent imaging work has taken a multivariate approach to language lateralisation, by investigating laterality measured across multiple language regions or language tasks within an individual (Berl et al., 2014; Bethmann, Tempelmann, Bleser, Scheich, & Brechmann, 2007; Häberling, Steinemann, & Corballis, 2016; van der Haegen, Cai, & Brysbaert, 2012; Tailby, Abbott, & Jackson, 2017; Woodhead, Bradshaw, Wilson, Thompson, & Bishop, 2019). Using fMRI, Häberling et al. (2016) compared a production with a comprehension task and found that although most participants showed concordance in their lateralisation across these tasks, there were a small number of individuals who showed crossed dominance. In a functional transcranial Doppler sonography (fTCD) paper, Woodhead et al. (2019) took laterality measurements across six language tasks within the same individuals, and fit a two‐factor model to the data using structural equation modelling. Although the majority of participants showed highly correlated scores on the two laterality factors, a small number showed uncorrelated scores indicating dissociated laterality across language functions. This study also confirmed that the dissociations were not explicable in terms of low test–retest reliability of laterality indices in specific tasks. This body of work indicated that different aspects of language can lateralise independently, requiring revision of the construal of lateralisation as a unitary construct (Bradshaw, Bishop, & Woodhead, 2017; Bradshaw, Thompson, Wilson, Bishop, & Woodhead, 2017).

In the light of such findings, the current paper tests an alternative hypothesis raised by Bishop (Bishop, 2013; Bishop et al., 2014) that consistency rather than degree of language lateralisation may constitute an aetiological risk factor for development of language impairment. Genetic accounts have suggested two underlying phenotypes for cerebral lateralisation: one with a left brain bias and one with no bias to left hemisphere language (Annett, 1985; McManus & Bryden, 1992). In the former, development of language laterality is biased towards the left hemisphere, for example with a weighting of 90:10; conversely, in the no bias phenotype, language laterality has equal likelihood of developing left or right bias. In the ‘Left Brain Bias’ model (Bishop et al., 2014), development of lateralisation for each aspect of language functioning is considered as an independent probabilistic process that occurs either with this systematic left bias or without any such bias. Consequently, this model predicts that individuals with the no‐bias phenotype will be more likely to show inconsistency in the side of lateralisation of different language functions. This could represent a less efficient language network, increasing the need for hemispheric integration and transfer of information across the corpus callosum.

In line with Bishop's model, it has previously been suggested that lateralised neural networks may have evolved in response to increases in callosal transmission delays associated with larger brains (Aboitiz, López, & Montiel, 2003; Ringo, Doty, Demeter, & Simard, 1994). This may have created an evolutionary pressure for functions that rely on multiple frequent interactions to be contained within the same hemisphere, so as to depend on relatively faster within‐hemispheric tracts. Similarly, Kosslyn (1987) argued that an innately specified leftward bias for a speech control centre leads to a snowball effect in which related functions will tend to develop congruent lateralisation. The implication of these ‘network efficiency’ models of lateralisation is that participants who fail to develop consistent lateralisation of different language processes would be expected to have less efficient language networks, resulting in impaired language function.

To our knowledge, *consistency* of language lateralisation has not previously been considered when investigating correlates of language impairment. The study by Wilson and Bishop (2018) measured lateralisation using a single animation description paradigm, while the fMRI study by Mellet et al. (2014) used a single production task and calculated laterality based on whole hemispheres. Thus, neither study was able to investigate potential dissociations in laterality at either a regional or language processes level. Indeed, the ‘weak laterality’ reported to be associated with poorer language functioning could in fact stem from independent lateralisation of frontal and posterior areas to different hemispheres within individuals, resulting in an LI that looks ‘bilateral’ when measured across whole hemispheres. A study by de Guibert et al. (2011) did measure laterality in a group of children with specific language impairment using a panel of four language tasks; however, these were simply used in a combined task analysis, without any comparison of individual patterns of laterality across the tasks.

### 1.3 Aims of the current study

This study aimed to test the novel hypothesis that inconsistent language laterality is associated with poorer language abilities. We used fTCD to measure lateralisation across three of the language tasks used by Woodhead et al. (2019) (phonological decision, semantic decision and sentence generation). The tasks were designed to engage distinct components of language functioning whilst being closely matched on non‐linguistic parameters.

There are potentially two ways of testing for an association between inconsistent lateralisation and language impairment. First, one can subdivide a sample according to consistency and compare their language abilities. Second, one can subdivide a sample according to whether or not they are language‐impaired and compare the frequency of inconsistent lateralisation. Although these address the same question, they may not always agree: Bishop (2013) suggested that studies adopting the first approach may fail to find associations if the sample contains only a small proportion of individuals with language problems. In the current study, we planned to adopt the first approach, by recruiting a heterogeneous sample with a high proportion of individuals with language impairment and measuring language abilities continuously. We categorised participants according to lateralisation consistency or inconsistency across three tasks, rather than by the presence or absence of a language impairment.

Because we aimed to recruit individuals with a range of language skills, we accepted into the sample individuals with a range of diagnoses. Diagnostic labels are not always informative for indicating extent and type of impairment and can often depend on multiple external factors such as the type of clinician giving the assessment (Astle, Bathelt, & Holmes, 2019). As such, we felt that an approach which measured language abilities continuously rather than relying on strict inclusion criteria with regard to previous diagnosis would likely be more informative.

We used a battery of standardised language tests to test differences in language ability between these consistent and inconsistent laterality groups. Our participant sample (*N* = 104) was designed to have a high degree of variability in language abilities; hence, we recruited an overrepresentation of language impaired individuals.

## 2 MATERIALS AND METHODS

This study was pre‐registered prior to data analysis on Open Science Framework (https://osf.io/fvhxq/register/565fb3678c5e4a66b5582f67). Deviations from this pre‐registration are described in the relevant sections of text and summarised at the end of the methods section.

### 2.1 Participants

A total of 109 participants were recruited and tested. In four of these participants, it was not possible to identify a robust signal from the middle cerebral artery during the fTCD procedure due to increased thickness of the temporal bone window. A further participant was excluded due to previous infection of the cerebellum. This left a total of 104 participants. This sample was made up of 36 male and 68 female individuals aged 18–46 years (mean age = 24.29 years), of whom 92 had typical right handedness and 12 had atypical handedness (11 left‐handed, one ambidextrous).The size of the sample was determined by the use of a power analysis, which will be outlined in a later section (see *Statistical Power Analysis*).

Within this sample, we aimed to recruit a high number of individuals with diagnoses of developmental disorders affecting language and communication, such as dyslexia, autism and dyspraxia. In total, 67 participants had such a diagnosis, termed the DD group (25 male, 58 right‐handed, mean age = 24.2 years), and 37 participants had no diagnosis, termed the control group (11 male, 34 right handed, mean age = 24.5 years). Recruitment was predominantly via disability services at local institutes of higher education for the DD group (including the University of Oxford, Oxford Brookes University, New Bucks University and City, University of London) and via an online participant recruitment system for the control group. The make‐up of the DD group in terms of diagnoses was as follows: 41 dyslexia, 10 dyspraxia/DCD, 10 dyslexia and dyspraxia/DCD, 4 autism and 2 specific learning difficulty (SpLD). Exclusion criteria for both groups were significant hearing loss, history of neurological disease, head injury or epilepsy. Most participants were current students enrolled on a university or college of higher education course (82 participants); only 8 participants (6 controls, 2 DD) had never been through higher education. This was therefore a high functioning sample. The two groups did not differ on a measure of non‐verbal reasoning (Cattell culture fair test, *t* = 0.06, *df* = 72.67, *p* = .952).

According to self‐report questionnaire data from the 67 individuals in the DD group, 52.2% of this sample had received special help or learning support while at school or university. Only 10.4% of the DD group reported having received speech and language therapy. A family history of similar problems was reported in just over half this group, with 53.7% reporting they had a family member with a history of speech, language, reading or communication disorder. The majority of the participants received their diagnosis while at school (65.7%), with the rest being diagnosed at university (except for one participant diagnosed through work).

Ethical approval for this study was granted by the Medical Sciences Inter‐divisional Research Ethics Committee (IDREC) at the University of Oxford (Approval Number R40410/RE001). The experiment was undertaken with the understanding and written consent of each participant.

### 2.2 Materials and procedure

Participants underwent two testing sessions; a first session in which a battery of language assessments was administered, and a second session in which functional transcranial Doppler sonography (fTCD) was used for laterality measurement. These sessions were either administered on the same day, or with a delay ranging up to 16 months apart (average delay = 12.8 weeks).

#### 2.2.1 Session 1: measurement of language skills

Session one lasted between one and two hours. Language skills were measured using a battery of standardised adult language assessments. These tests yielded 12 language measures per participant. The tests included and standardised mean scores of the DD and control groups (based on test standardisation norms) are given in Table 1. Note that one of these measures was changed from that pre‐registered; TOWRE non‐word reading was replaced by TOWRE overall standard score due to ceiling effects in the former. In addition to these language measures, some handedness measures were included, only one of which is reported here: a quantitative measure of handedness from the Annett peg moving task (Annett, 1985). Tests one and two of the Cattell Culture Fair Test (Cattell & Cattell, 1973) were also administered in order to obtain a measure of non‐verbal reasoning.

**Table 1:** Language assessments. The 12 measures used to obtain language ability scores. Mean standardised scores (and standard deviations, *SD*) based on test standardisation norms are given for each measure for DD and control groups. Note that norms for NEPSY measures were calculated using scores from an adult sample reported by Barry, Yasin, and Bishop, (2007), since the test only provides standardisation based on a sample of children

Instrument	Measure(s)	Domain being tested	DD group mean standard score (*SD*)	Control group mean standard score (*SD*)
Expression, Reception and Recall of Narrative Instrument (ERRNI) (Bishop, 2004)	1. Mean length of utterance (MLU)	Expressive language	102.31 (13.45)	109.86 (13.09)
	2. Story comprehension	Comprehension (non‐reading based)	108.64 (13.18)	110.59 (12.84)
Wechsler Adult Intelligence Scale (WAIS) (Wechsler, 1997)	3. Digit span (total score)	Phonological short‐term memory and working memory	9.78 (2.76)	11.57 (2.23)
Wechsler Abbreviated Scale of Intelligence (WASI) (Wechsler, 1999)	4. Vocabulary	Verbal IQ	61.54 (7.33)	62.39 (7.22)
York Adult Assessment Battery‐ Revised (YAA‐R) (Warmington, Stothard, & Snowling, 2013)	5. Rapid naming objects	Rapid naming	1.74 (0.36)	2.04 (0.24)
Test of Word Reading Efficiency (TOWRE) (Torgesen, Wagner, & Rashotte, 1999)	6. Overall reading standard score	Word and non‐word reading	92.73 (16.17)	108.19 (11.19)
NEPSY‐A developmental neuropsychological assessment (Korkman, Kirk, & Kemp, 1998)	7. Oromotor sequences	Articulation	61.61 (5.05)	63.41 (3.79)
	8. Non‐word repetition	Phonological short‐term memory	40.72 (4.03)	42.32 (3.52)
Test of Adolescent and Adult Language (TOAL−4) (Hammill, Brown, Larson, & Wiederholt, 2007)	9. Sentence assembly	Syntax	6.81 (1.89)	7.24 (1.61)
Communication Checklist self‐report (Bishop, Whitehouse, & Sharp, 2009)	10. Language structure	Language structure	8.29 (3.46)	10.22 (3.89)
	11. Pragmatics	Pragmatic skills	9.37 (3.79)	10.32 (3.02)
	12. Social engagement	Social engagement	9.88 (3.99)	11.49 (2.80)

Data collected using these language measures was stored on REDCap electronic data capture tools (Harris et al., 2009) hosted at Oxford University. REDCap (Research Electronic Data Capture) is a secure, web‐based application designed to support data capture for research studies, providing: (a) an intuitive interface for validated data entry; (b) audit trails for tracking data manipulation and export procedures; (c) automated export procedures for seamless data downloads to common statistical packages; and (d) procedures for importing data from external sources.

#### 2.2.2 Session 2: measurement of lateralisation

In session two, lateralisation of language function was measured using functional transcranial Doppler ultrasound (fTCD). FTCD is a reliable method of laterality measurement which uses ultrasound waves to insonate the middle cerebral artery (MCA) of each hemisphere in order to measure changes in the speed of blood flow in right and left hemispheres (Deppe, Knecht, Henningsen, & Ringelstein, 1997; Knecht et al., 1996). Participants were first set up with the fTCD headset and probes, before performing each language task in a randomised order. All stimuli were presented using Psychtoolbox (Brainard, 1997) for Matlab 2012a (Mathworks Inc, Natick, MA). Each task lasted around 15 min, giving a total session time of 1–1.5 hr depending on the length of time required for fTCD set‐up.

**Figure 1:**
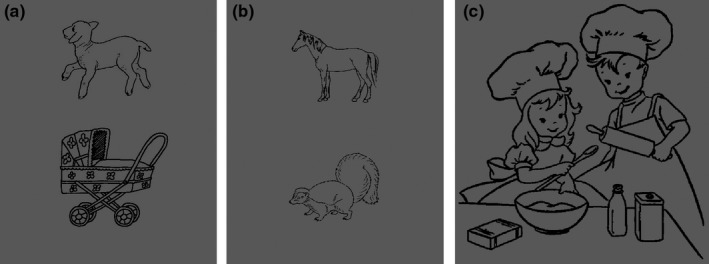
Example picture stimuli used in the three tasks. From left to right: (a) phonological decision, (b) semantic decision and (c) sentence generation. The majority of the picture stimuli were taken with permission from the International Picture Naming Project (Szekely et al., 2004)

The following fTCD tasks were selected from a larger task battery reported on by Woodhead et al. (2019): phonological decision (PD), semantic decision (SD) and sentence generation (SG). These tasks were reported by this paper to have moderate to very good test–retest reliability (PD: *r* = .54, SD: *r* = .74, SG: *r* = .84). The procedure of these tasks is outlined in detail in Woodhead et al. (2019). Briefly, the two decision tasks involved making yes/no button press decisions on pairs of picture stimuli, based on either whether the objects belonged to the same semantic category (semantic decision) or whether the words represented by the pictures rhymed or not (phonological decision). Sentence generation required participants to covertly generate and then overtly report a sentence to describe each of a series of presented pictures. Example stimuli for the three tasks can be found in Figure 1.

**Figure 2:**
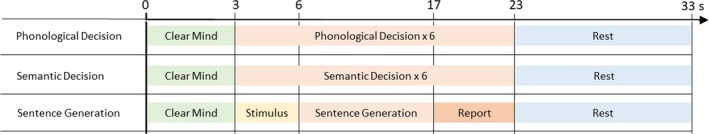
FTCD tasks. Task design and timings of the three language tasks for use with fTCD [Colour figure can be viewed at http://wileyonlinelibrary.com]

The tasks were designed to engage distinct aspects of language processing while minimising differences in engagement of non‐linguistic processes that may contribute to differences in the measured LIs. All tasks were cued by similar black line drawing stimuli, in order to closely match perceptual processing across tasks. In particular, the two decision tasks used highly similar sets of picture stimuli representing words that were matched across task sets on the following psycholinguistic variables using N‐watch: familiarity, orthographic neighbourhood, imageability, number of phonemes and frequency. All three tasks were also designed so as to follow a similar structure (illustrated in Figure 2). Each task was made up of 15 trials, each 33 s long. Trials started with the word ‘CLEAR’ on screen for 3 s, instructing participants to clear their mind in preparation for the next trial. This was followed by a 20‐s period during which the task is performed. After the task period for that trial had finished, the word ‘REST’ appeared on screen for 10 s. Participants were instructed to try and keep a clear mind during this rest period.

### 2.3 FTCD analysis and calculation of laterality indices

The fTCD data were analysed using a custom R script (this can be found at: https://osf.io/k7nhz/). Details of this analysis have been described in a previous paper (Woodhead et al., 2019). Briefly, after various pre‐processing stages to normalise the signal in left and right channels, a left minus right difference curve averaged across trials within a task was calculated for each individual. These curves were then used to calculate an individual's laterality index (LI) for each task. The ‘mean method’ of LI calculation was used in which the LI was taken as the mean of the averaged left minus right differences within a period of interest (between 6 and 26 s peristimulus time for the decision tasks, and between 6 and 20 s for the sentence generation ask). Note that this method differs from the classic ‘peak method’ of LI calculation that was originally pre‐registered. The mean method was preferred as it results in LI values with a more normal distribution and is more robust to outlying observations (e.g. artefactual spikes in one channel's signal). This change in LI calculation method did not change the outcome of analyses.

The resulting LI values were used to classify participants into two dominance categories for each task, left lateralised (L) or right lateralised (R), according to whether their LI value was positive or negative, respectively. It was decided not to include bilateral as a dominance category due to concerns as to the reliability of this classification (see Jansen et al., 2006 and Bradshaw, Bishop, et al., 2017, for discussion of this in the fMRI literature). A participant was classed as ‘consistent’ if all three of their dominance classifications for the different tasks agreed. A participant was classed as ‘inconsistent’ if there was discordance in their dominance classification for the three tasks.

### 2.4 Statistical analyses

All analyses were conducted using the statistical software R (R Core Team, 2019). The R Markdown script used to run all analyses can be found on Open Science Framework (https://osf.io/k7nhz/).

#### 2.4.1 Pre‐registered analyses

The main analysis of this study tested the hypothesis that inconsistent lateralisation across the three fTCD tasks would be associated with poorer language skills. The whole sample (composed of both developmental disorder and control individuals) was divided into those with consistent and inconsistent laterality, that is those with the LI on the same side for all three tests versus the remainder. These two groups were then compared on the 12 language ability measures using a multivariate test. The original pre‐registered analysis plan was to use the multivariate counterpart of the *t* test, the Hotelling's T‐square test (see Schumacke, 2016). However, due to non‐normality of the language measure data, a nonparametric multivariate test was used instead to compare the language measures between laterality groups (Oja & Randles, 2005; Sirkiä, Taskinen, Nevalainen, & Oja, 2007). This test can be considered as a multivariate extension of the Wilcoxon–Mann–Whitney test, but with altered calculation of signs and ranks to reflect the multidimensionality of the data. The test statistic, *Q^2^*, follows a chi‐squared distribution and thus can be compared with the chi‐squared critical value to test for significance. This nonparametric multivariate test was run using the R package SpatialNP (Sirkiä et al., 2007). We predicted that this would be significant, indicating that the two groups significantly differed on these measures as a set.

As a positive control to establish that our language measures were sensitive to language impairments, we planned to re‐run this multivariate analysis comparing the 12 language measures between the DD and control groups. We predicted that this would yield a significant result in which the scores of the DD group would be significantly lower than those without a diagnosis.

To investigate the potential relationship between handedness and consistency of language lateralisation, strength of right handedness was compared between consistent and inconsistent groups measured using the Annett peg moving task. This uses speed of performance with the right and left hand (to move wooden pegs from one row of a peg board to the other) to calculate a handedness quotient of ((L − R)/(L + R)*100). This normally distributed measure of relative hand skill was compared between the consistent and inconsistent groups by means of an independent *t* test, with the prediction that the inconsistent group would demonstrate lower relative right hand skill than the consistent group.

#### 2.4.2 Non‐preregistered exploratory analyses

Further analyses that were not pre‐registered were carried out on the data. Pearson's correlations were used to explore the relationships between LIs from different tasks. This was performed for the DD and control groups separately to test the non‐preregistered hypothesis that correlations between task LIs would be weaker in the DD group than the control group. Further, to investigate whether the DD group demonstrated a significantly increased frequency of inconsistent laterality patterns, a chi‐squared test was used to compare frequencies in the inconsistent and consistent laterality groups from the two diagnosis groups.

One possibility is that individuals with developmental disorders may not only be inconsistent in which hemisphere they use across different language tasks, but also across time within performance of a single task. To investigate this, the percentage of trials within each task that were left lateralised was calculated for each individual (e.g. a score of 0% of trials would indicate consistent right lateralisation; 50% would indicate maximum inconsistency; and 100% would indicate consistent left lateralisation). A metric of consistency in lateralisation over time was then obtained by taking the absolute difference between this value and 50. This consistency metric could therefore range from 0 to 50, where 0 indicates maximal inconsistency (50/50 split in terms of which side was used across trials), and 50 indicates complete consistency (one side was used on all trials). Mean values of the consistency metric were then compared between control and DD groups for each task using independent *t* tests, with the exploratory hypothesis of a reduced level of consistency over time in the DD group.

Lastly, although the main hypothesis of this study focused on the relationship between language abilities and consistency in lateralisation, the data were further analysed in a more conventional way to test for a relationship with strength of lateralisation. A multi‐level modelling analysis was used to compare strength of laterality between the DD and control groups across the three laterality tasks.

### 2.5 Statistical power analysis

The pre‐registered power analysis to determine sample size was based on the use of the Hotelling's T‐square test, as originally planned. This first required an estimate of effect size. To our knowledge, there is no previous literature using this kind of analysis to look at the relationship between consistency in lateralisation and language ability. A moderate effect size of *d* = 0.5 was therefore chosen, which represents the size of effect which would be of theoretical interest. On the basis of previous work using these tasks (Woodhead et al., 2019), it was predicted that the numbers of participants falling into consistent versus inconsistent laterality groups would follow a ratio of 2:1. Based on this effect size and ratio of participants in the two groups, a power level of 0.9 using 12 language measures yielded a required sample size of 65 for the consistent laterality group and 33 for the inconsistent laterality group.

### 2.6 Summary of departures from pre‐registered methods

To summarise, the following changes were made from the pre‐registered protocol:

1. Change in one of the 12 language measures. The pre‐registered measure of TOWRE non‐word reading was replaced by TOWRE overall standard score due to ceiling effects in the former.
2. Laterality index calculation method. The pre‐registration stated that the peak method of LI calculation would be used, as is conventional in fTCD analyses. However, as outlined above, the decision was made to use a newly developed mean method of LI calculation, as this provides a more representative measure of laterality for the individual, and a more normally distributed measure at the group level.
3. Choice of multivariate test to compare the consistent and inconsistent laterality groups on language measures. The pre‐registered analysis to compare laterality groups on the 12 language measures used the parametric Hotelling's T‐square test. However, since data on the language measures were found to be non‐normal, a nonparametric equivalent of this multivariate test was used for this comparison.

## 3 RESULTS

### 3.1 Task LI values

**Figure 3:**
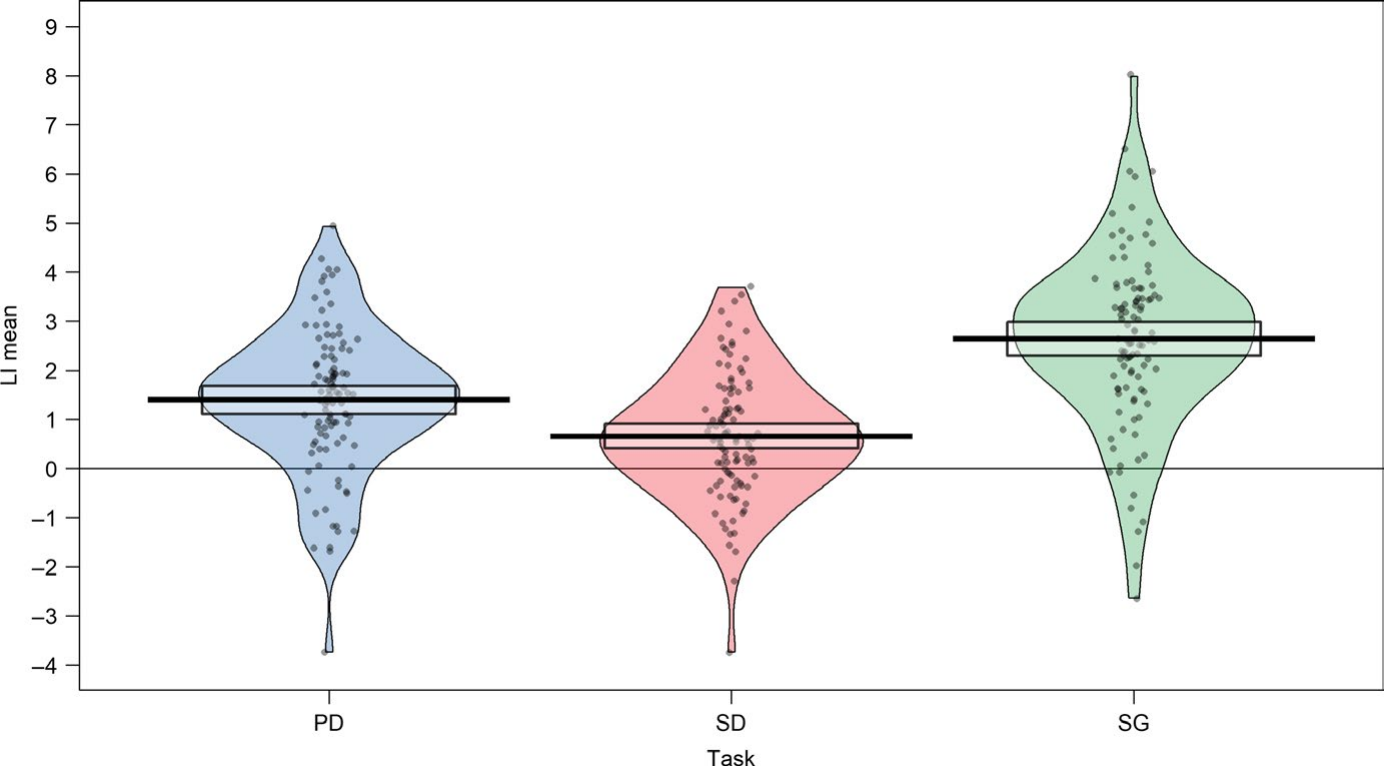
Mean method of LI calculation. LI values across the whole sample for each task, calculated using the mean method. Bars indicate the mean and shaded boxes 95% confidence intervals. PD = phonological decision, SD = semantic decision, SG = sentence generation [Colour figure can be viewed at http://wileyonlinelibrary.com]

LI values on each of the three tasks are given in Figure 3. All three tasks demonstrated significant left lateralisation at the group level (95% confidence intervals do not contain zero).

### 3.2 Pre‐registered analyses

#### 3.2.1 Consistency in lateralisation

Within the sample, 31 individuals were identified with inconsistent lateralisation across the three tasks and 73 individuals with consistent lateralisation (collapsing across diagnosis groups). For a small number of participants (four individuals), classifications into consistent and inconsistent groups based on peak and mean LI values did not agree. This meant that although the required sample size of 33 in the inconsistent group was reached based on peak LI classifications, only 31 participants were classified as inconsistent using the mean measure. This small reduction was considered still acceptable given the power calculation that yielded these required sample sizes was based on a high power level of 0.9.

The most common pattern in the inconsistent laterality group was left lateralised for PD and SG and right lateralised for SD (21/31 individuals). In general, SD was more likely to dissociate from the other two tasks; in contrast, PD and SG tended to lateralise together, especially if PD was left lateralised.

#### 3.2.2 Comparison of language measures between consistent and inconsistent groups

**Figure 4:**
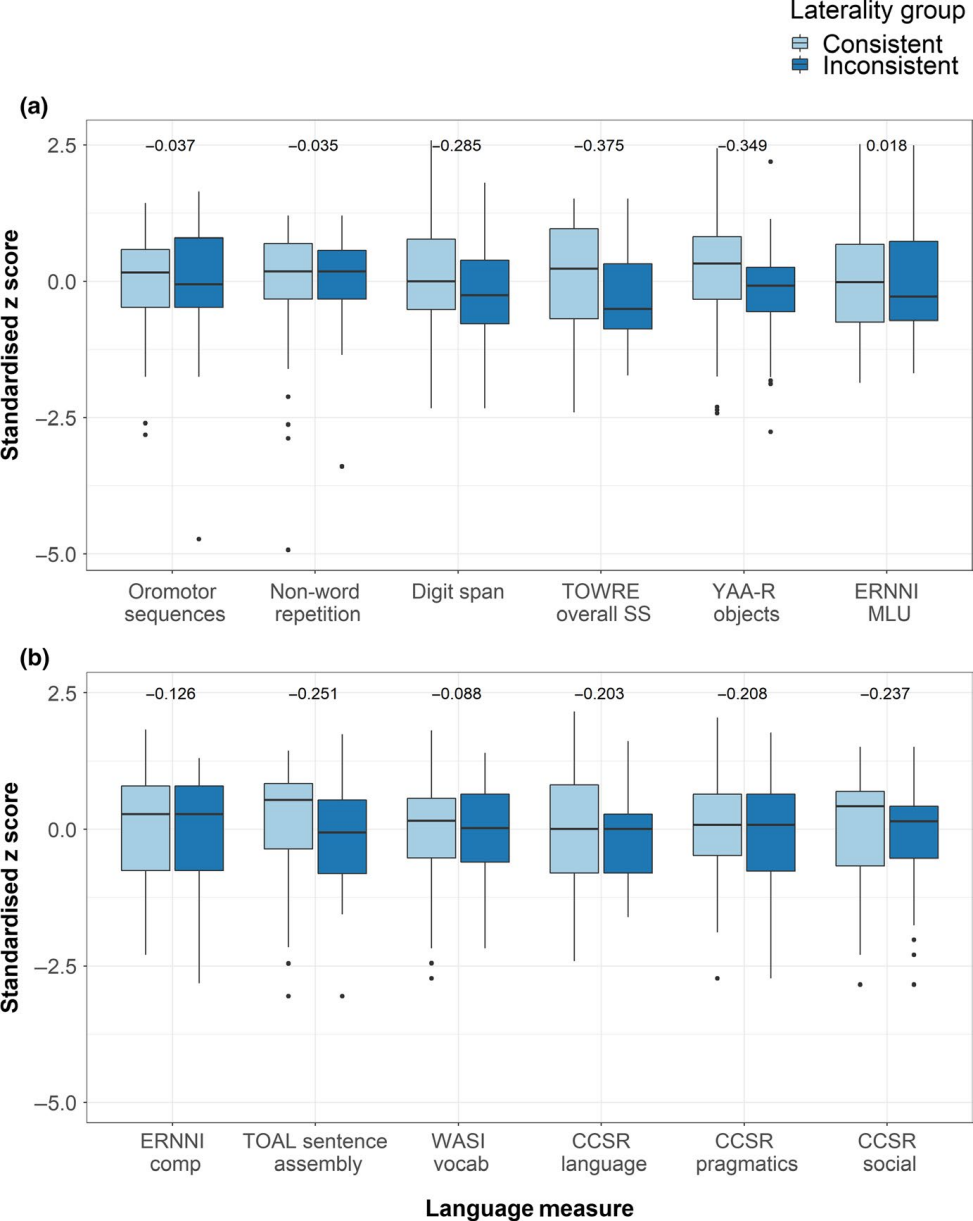
Laterality group language performance. Boxplots of standardised scores on each of the 12 language measures in the consistent and inconsistent laterality groups. The first six measures are shown in panel A, and the last six measures in panel B. The values above pairs of boxplots indicate standardised (z‐score) differences between the means of the two laterality groups for each measure (negative values indicate better performance of the consistent group) [Colour figure can be viewed at http://wileyonlinelibrary.com]

For data visualisation, all scales were transformed to z‐scores based on the mean and SD of the whole sample to facilitate comparison across measures. Standardised scores in consistent and inconsistent laterality groups for each of the 12 language variables are given in Figure 4 as boxplots. A multivariate nonparametric test found no statistically significant difference between groups across the measures (*Q* ^2^ = 6.49, *df* = 12, *p* = .889). This indicates that the inconsistent and consistent groups did not differ in their language abilities, meaning the main hypothesis of the study was not confirmed. Means and standard deviations for consistent and inconsistent groups on these 12 measures can be found in the supporting information for this paper (Table S1).

This multivariate analysis was repeated as a positive control, comparing DD and control groups on the same 12 language measures. This did yield the expected significant group difference (*Q* ^2^ = 34.06, *df* = 12, *p* < .001), indicating that the language measures were sensitive enough to distinguish between the DD and control groups. Means and standard deviations for DD and control groups on these 12 measures can be found in the supporting information for this paper (Table S2).

#### 3.2.3 Consistency in lateralisation and handedness

An independent *t* test found no significant difference in right handedness as measured by the Annett peg moving task between consistent (*M* = 2.33, *SD* = 4.04) and inconsistent (*M* = 2.30, *SD* = 3.26) groups, *t*(69.71) = 0.045, *p* = .965, *d* = 0.009.

### 3.3 Non‐preregistered exploratory analyses

#### 3.3.1 Correlations between task LIs

**Figure 5:**
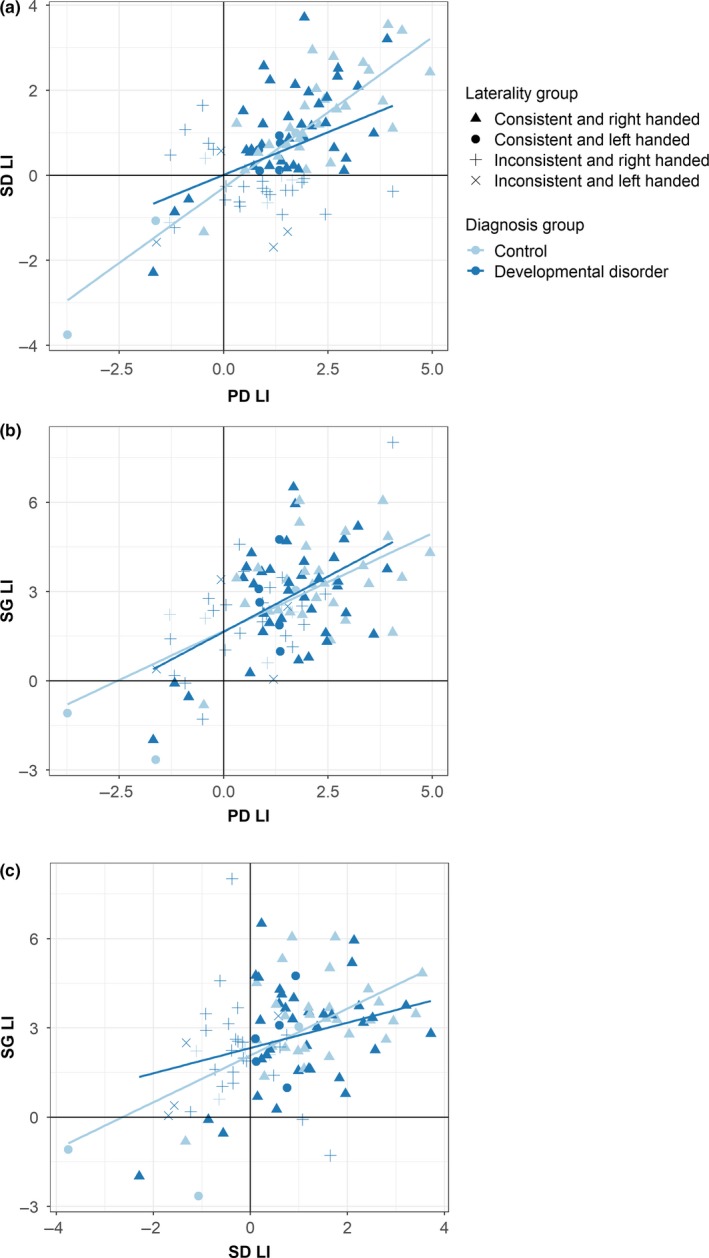
Scatter plots showing relationships between pairs of task LIs: (a) Phonological decision and semantic decision, (b) Phonological decision and sentence generation, and (c) Sentence generation and semantic decision. Regression lines are fit separately for each group (control vs. DD). Different symbols indicate handedness of participants (right or left) and consistency of laterality (consistent or inconsistent) [Colour figure can be viewed at http://wileyonlinelibrary.com]

Scatter plots of LI values were used to further explore patterns of inconsistent lateralisation across the tasks (see Figure 5). Discrepant laterality between two tasks is indicated by the presence of points in the upper left and lower right quadrants of these plots. Most striking is the absence of points in the lower right quadrant of the PD‐SG plot, indicating that no individuals demonstrated leftward lateralisation for PD but rightward lateralisation for SG.

Pearson's correlations (for the DD and control groups separately) can be found in Table 2. All correlations were significant (using a Bonferroni corrected *p* value of .008) except that between SG and SD for the DD group. The correlations for the DD group were all weaker than those for the control group, particularly for the PD–SD correlation, where the 95% confidence intervals for the two groups did not overlap. In other words, the DD participants had less consistent laterality strength across tasks than controls.

**Table 2:** LI correlations in diagnosis groups. Pearson's correlations and 95% confidence intervals (CI) for pairs of tasks in DD and control groups. PD = phonological decision, SD = semantic decision, SG = sentence generation

	Dev. disorder group *r*	95% CI	Control group *r*	95% CI
PD_SD	.437	0.22–0.61	.854	0.73–0.92
PD_SG	.537	0.34–0.69	.636	0.39–0.79
SG_SD	.282	0.04–0.49	.632	0.39–0.79

#### 3.3.2 Consistency of lateralisation in the two groups

A summary of the numbers in each laterality group from the DD and control groups is given in Table 3. A chi‐squared test found that the frequency of consistent versus inconsistent lateralisation in the DD and control groups was significantly different from those that would be expected by chance (*χ* ^2^ = 7.29, *p* = .007, *df* = 1, *ϕ* = 0.265). This was driven by a higher incidence of inconsistent lateralisation in the DD group compared to the control group.

**Table 3:** Frequencies of consistent and inconsistent laterality cases in each diagnosis group

Group	Consistent laterality	Inconsistent laterality
Developmental disorder	41	26
Control	32	5

To check that such group differences could not be attributable to differences in behavioural performance on the fTCD tasks, performance measures were compared between DD and control groups. Mean accuracy and reaction times are given in Table 4 for each group. Independent *t* tests found no significant differences between these two groups on any of the behavioural measures (lowest *p* value = .255). Comparisons between the two decision tasks across the whole group did however find significant differences in accuracy (*t*(103) = −7.287, *p* < .001, *d* = 0.74), which was lower in the PD task (*M* = 0.89, *SD* = 0.07) than the *SD* task (*M* = 0.94, *SD* = 0.05); and also in reaction times (*t*(103) = 28.368, *p* < .001, *d* = 2.14), which were slower in the PD task (*M* = 1.72, *SD* = 0.22) than the *SD* task (*M* = 1.21, *SD* = 0.25). Correlation analyses found no significant relationships between task performance measures and LI values (*p *> .3 in all cases).

**Table 4:** FTCD task performance measures. Means (and standard deviations) for performance measures across the three fTCD tasks for each group

Group	PD accuracy (%)	PD RT	SD accuracy (%)	SD RT	SG number of words
Control	89.9 (5.34)	1.73 (0.19)	94.3 (6.16)	1.18 (0.22)	9.61 (0.81)
Developmental disorder	88.7 (8.39)	1.71 (0.23)	93.9 (5.07)	1.23 (0.26)	9.65 (1.39)

#### 3.3.3 Consistency in lateralisation over time

**Figure 6:**
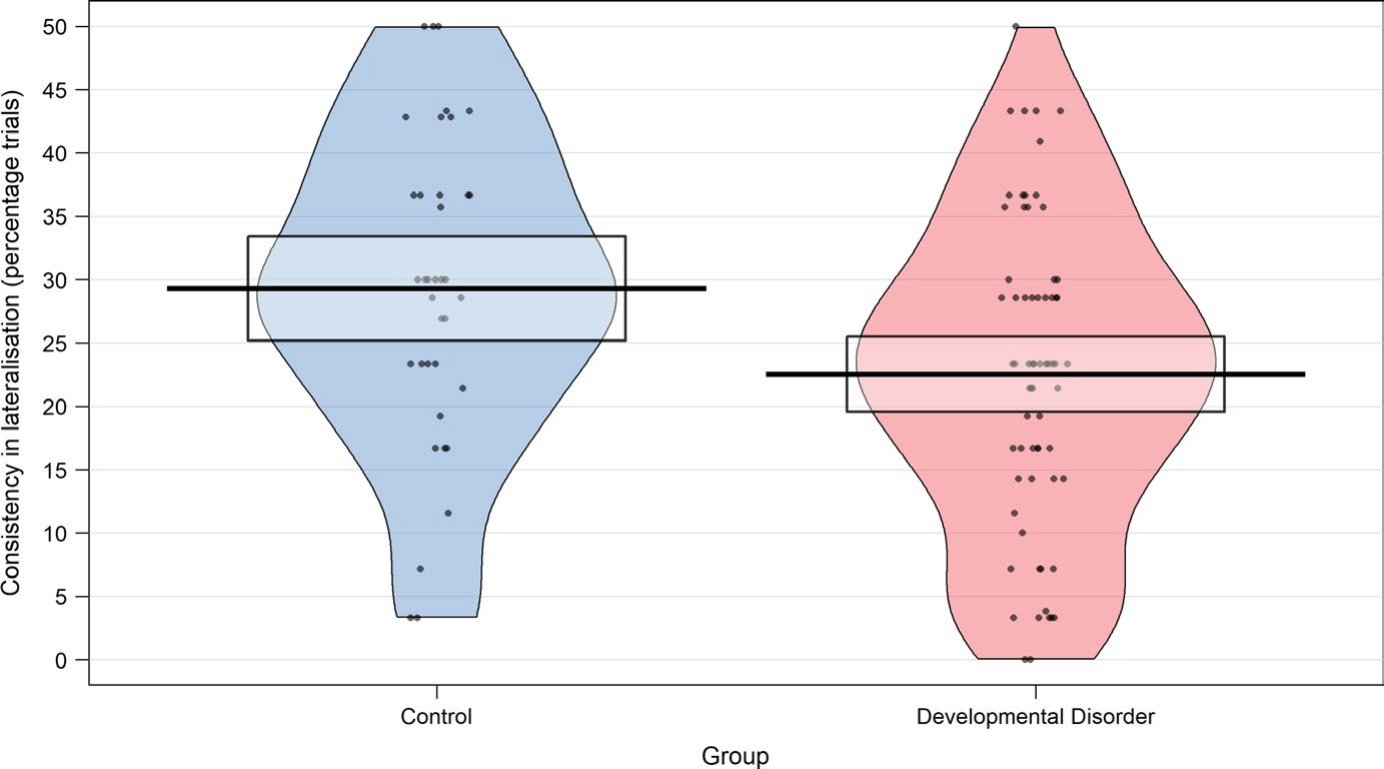
Consistency in lateralisation over time. Consistency in lateralisation over trials within the phonological decision task, for control and developmental disorder groups. Consistency in lateralisation was calculated as the absolute difference between 50 and the percentage of trials lateralised to the left. Higher values thus indicate greater consistency [Colour figure can be viewed at http://wileyonlinelibrary.com]

To investigate whether the group with developmental disorders demonstrated inconsistency in lateralisation from trial to trial within performance of a single task, the two groups were compared on a metric of lateralisation consistency across trials for each task individually (see *Methods: Non‐preregistered exploratory analyses*). Scores of the two groups on this metric can be found in Figure 6. Using a Bonferroni‐corrected *p* value of .0167, no significant group differences were found for SD or SG; however, a significant group difference was found for PD (*t*(73.35) = 2.69, *p* = .009, *d* = 0.55), with the DD group (*M* = 22.53, *SD* = 12.16) demonstrating reduced consistency in lateralisation across trials compared to the control group (*M* = 29.31, *SD* = 12.36).

#### 3.3.4 Comparing strength of lateralisation

**Figure 7:**
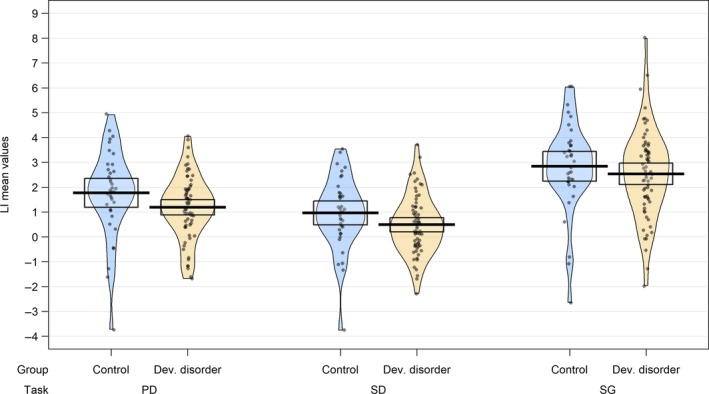
Strength of laterality. LI values (calculated using the mean method) in developmental disorder and control groups for phonological decision (PD), semantic decision (SD) and sentence generation (SG) tasks [Colour figure can be viewed at http://wileyonlinelibrary.com]

Multi‐level modelling was used to compare strength of laterality between DD and control groups across the three laterality tasks. LI values (calculated from the mean method) in each group across the three tasks are shown in Figure 7.

A multi‐level mixed model analysis was conducted using the *lme4* package in R (Bates, Mächler, Bolker, & Walker, 2015). This entered group and task as fixed effects (without interaction) and participant as a random effect into a random intercept, fixed slope model. This model found no significant effect of group on LI values (*β* = −0.451, *t* = 1.753, *p* = .083, *df* = 102). This did however find a significant effect of task; using PD as a reference condition, LIs were found to be significantly lower for SD (*β* = −0.738, *t* = −5.08, *p* < .001, *df* = 206), but significantly higher for SG (*β* = 1.248, *t* = 8.60, *p* < .001, *df* = 206).

## 4 DISCUSSION

### 4.1 Summary of results

This study aimed to test the pre‐registered hypothesis that inconsistent lateralisation across different language tasks within individuals is associated with impaired language skills. This hypothesis was not supported; a multivariate test found no significant difference between consistent and inconsistent laterality groups across the 12 language measures. Further exploratory tests did, however, show some suggestions that the developmental disorder group showed less consistent laterality across tasks than the control group: they had weaker correlations between tasks, and a higher incidence of inconsistent laterality patterns. Further, they demonstrated reduced consistency in lateralisation over time within performance of the phonological decision task. They did not however show significantly weaker laterality than the control group in a multi‐level modelling analysis with the three tasks.

### 4.2 Inconsistent laterality and language impairment

The results, then, appear quite paradoxical, with a rather different picture depending on how the analysis is conducted. The pre‐registered analysis, in which individuals were categorised by consistency of language laterality and compared on language measures, did not reveal any evidence for poorer language skills in those who were inconsistent. Our sample included a large proportion of individuals with developmental disorder, so the lack of a difference could not be attributed to a low base rate making it hard to detect an effect. Nevertheless, when we conducted an exploratory analysis that turned the question around and looked at laterality in those with developmental disorders, we did find evidence for an association. Indeed, only five of the 31 individuals with inconsistent laterality were in the control group. We also established that our language test battery was sensitive to developmental disorder, so the lack of an effect in the pre‐registered analysis could not be attributed just to test insensitivity. Overall, the pattern of results suggested that, while inconsistent laterality was predictive of developmental disorder, nevertheless, the majority of people with developmental disorder had consistent laterality.

The most obvious way to interpret this pattern is to conclude that developmental disorder is heterogeneous, with different neurobiological causes in different people. On this view, inconsistent laterality is just one factor that can impair language development, but its impact may be hard to detect because most cases of developmental disorder arise from other causal factors. We need also to be careful about concluding that we have completely characterised inconsistent laterality. We used just three lateralised language tasks with fTCD: it is possible that a different and/or larger set of tasks would have revealed more cases of inconsistency. A further possibility is that the correlate of inconsistent laterality is not poor language skills as such, but some other trait that is associated with a diagnosis of developmental disorder, such as attentional problems. Alternatively, inconsistent lateralisation and language difficulties may be traits that share common causes without themselves being causally related; for example, in a pleiotropy model, the same genes that increase risk for language problems could also increase risk for a disruption to lateralisation (Bishop, 2013; Paracchini, Diaz, & Stein, 2016). This account would thus predict an increased co‐occurrence of these traits, but allow for instances in which one can occur without the other due to the lack of direct causal relation.

The pattern of results is also consistent with the idea that it may be the experience of having a developmental disorder that increases the likelihood of development of an unusual organisation of the language network. Bishop (2013) referred to this possibility as the ‘neuroplasticity’ model, in which lateralisation has no causal implications for language, but language impairment itself has consequences for how the brain develops. For example, it has been proposed that establishment of lateralised circuits is driven in part by increasing proficiency and skill in processing language over the early years of life (Minagawa‐Kawai, Cristià, & Dupoux, 2011). One could extend this theory to predict that in cases of impaired language, reduced proficiency and slower rate of development of language skill may result in incomplete or weaker establishment of a specialised left hemisphere language system. Experience of impaired development of language skills could therefore impact lateralisation either by triggering attempts at compensatory reorganisation or in fact by failing to engage normal mechanisms that drive the establishment of left lateralised processing circuits for language. Overall, the pattern of results suggests the need to reconsider models of the relationship between language impairment and language lateralisation, including the direction of causality.

### 4.3 Patterns of laterality across language tasks

By taking laterality measurements across different language tasks within the same participants, the current study allowed for observation of different multivariate patterns of laterality within individuals. By far, the most common pattern of inconsistent laterality was left lateralisation for phonological decision (PD) and sentence generation (SG), but right lateralisation for semantic decision (SD). PD and SG showed particularly high concordance, with no cases of left laterality for PD but right laterality for SG. This may reflect the common engagement of subvocal rehearsal by both tasks, which was not required for SD. The frequent dissociation of PD laterality from SD laterality is however particularly striking given that this pair of tasks was designed to be as closely matched on non‐linguistic parameters as possible.

The dissociation between PD and SG versus SD may reflect the differing involvement of dorsal and ventral language streams, respectively (Hickok & Poeppel, 2007). SG required subvocal rehearsal of a generated sentence while PD required covert naming of the two pictures in order to make the rhyme judgement. These tasks are therefore likely to engage left posterior inferior frontal cortex (pars opercularis), which is important for subvocal rehearsal (Price, 2012).

Conversely, the SD task is considered a temporal task, typically engaging bilateral regions in the temporal lobe in fMRI studies (see Bradshaw, Thompson, et al., 2017 for a review). A meta‐analysis by Rice, Lambon‐Ralph, and Hoffman (2015) suggested that while semantic knowledge is represented bilaterally in the anterior temporal lobes, access to this information during linguistic processing was more likely to be left lateralised. While performance of the SD task encouraged linguistic access to semantic concepts, performance did not require explicit naming of the pictures in the same way as the PD task; it is possible therefore that variability in the strength of left lateralisation of this task may reflect individual variability in engagement of a linguistic‐based strategy for performance of the task.

### 4.4 Inconsistency in lateralisation over time

Language lateralisation is traditionally seen as a fixed characteristic of the individual. Our final exploratory analyses, however, suggested that people may vary in how variable they are in engaging the two hemispheres in a task. We demonstrated that the developmental disorder group appeared to engage in more frequent switching between dominance of the two hemispheres during the PD task. The developmental disorder group did not demonstrate significantly worse performance on the PD task relative to the control group, but it is likely that this phonological task was particularly challenging for the high number of dyslexic participants in this sample. We may speculate that these participants could have recruited additional right hemisphere resources in order to compensate for deficient left hemisphere phonological processing. This more frequent switching between hemispheres during performance of the PD task may therefore reflect a compensatory mechanism in the current high‐functioning sample. It would be of interest to use fMRI to further investigate this hypothesis and specify which right hemisphere areas may be recruited by these individuals; in theory, these could either be the right homologues of the left language areas or domain‐general areas.

### 4.5 Limitations of the study

This study tested a sample of high functioning adults with a range of developmental disorders. Although these disorders are typically associated with impairments in language skills, most participants were students enrolled on a university course and thus did not demonstrate severe impairments. It could be argued that a more clinically impaired or a child sample would have provided a better context in which to test this hypothesis. Nevertheless, we were able to show that the developmental disorder group was significantly impaired across a range of language measures compared to control participants. Thus, the failure to find evidence for a difference in language abilities between consistent and inconsistent laterality groups cannot be attributed to a lack of a range of language ability levels in the sample.

The benefits of the fTCD method must be weighed against a number of limitations when compared to other imaging methods. Measurement of lateralisation with fTCD is not only limited by a lack of regional specificity, but is also constrained by the territory of the middle cerebral artery. Although this covers many key areas within the language network, one region outside this territory is the ventral occipitotemporal cortex (vOT), often termed the ‘visual word form area’. Interestingly, fMRI work by Tailby, Weintrob, Saling, Fitzgerald, and Jackson (2014) in patients with epilepsy suggests this region may demonstrate important differences in laterality according to language abilities. They reported that laterality in vOT was important for distinguishing epilepsy patients with reading disorders from those without, with the former demonstrating greater bilaterality in this area. VOT has been demonstrated to co‐lateralise with frontal expressive language regions in healthy controls (van der Haegen et al., 2012; Seghier & Price, 2013). It is possible therefore that dissociation of laterality of this posterior area from inferior frontal language areas may constitute a risk factor for reading impairment. Such dissociations would likely go undetected by the fTCD method, since vOT is on the boundary of the MCA/posterior cerebral artery territory (Kim et al., 2019). FMRI would enable testing of the hypothesis that inconsistent lateralisation across inferior frontal and ventral occipitotemporal regions is associated with reading difficulties.

This study constitutes the first to our knowledge to investigate the relationship between language impairment and lateralisation with reference to the multidimensionality of lateralisation across different language functions. As such, the use of the cheaper and more quickly administered fTCD method to a large sample was considered a useful first step in testing for developing research hypotheses that can then be followed up by more expensive and time intensive fMRI investigations.

## 5 SUMMARY AND CONCLUSIONS

This study did not find evidence that inconsistent laterality across different language tasks (indicative of a more distributed language network) was associated with poorer language skills as hypothesised. However, exploratory analysis of our data does suggest that such inconsistent profiles of language laterality may be more likely in individuals with developmental disorders, and indeed, it was unusual to find a control participant with inconsistent laterality. The results go against the notion of inconsistent lateralisation as a major cause of developmental language impairment, but nevertheless suggest it may be a contributory factor in a subset of individuals. We caution, however, against assuming a specific direction of causality to explain this association. Our results are also consistent with pleiotropy, that is a common causal factor that independently increases risk of language impairment and inconsistent lateralisation, or a neuroplasticity model, in which experience of a developmental disorder could lead to changes in brain organisation for language. Overall, the results of the current work highlight the importance of taking a multivariate approach to the study of language laterality. Measurement with only one task does not provide a sufficient description of individual variability in laterality patterns, and so is not appropriate for investigation of the significance of such individual variability to language functioning.

## CONFLICT OF INTEREST

The authors declare no conflict of interests.

## AUTHOR CONTRIBUTIONS

ARB designed the study, analysed data and drafted the paper. ZVJW helped to design study, and reviewed and edited the manuscript. PAT contributed to statistical analysis of the data. DVMB conceived the experiment, and reviewed and edited manuscript.

## Data Availability

All analysis scripts and anonymised data are available on Open Science Framework (https://osf.io/k7nhz/).
