# Supplementary material for: Investigation into inconsistent lateralisation of language functions as a potential risk factor for language impairment
Source: Eur J Neurosci. 2019 Dec 14;51(4):1106–21. doi: 10.1111/ejn.14623 (PMC7078955; doi:10.1111/ejn.14623)
Supplement: Supplementary file 1 [file EJN-51-1106-s001.pdf]

Supporting Information for ‘Inconsistent lateralisation of language functions: a risk factor for language impairment?

Supplementary Table 1: Means and standard deviations (SD) for consistent and inconsistent laterality groups on each of the 12 language measures. Effect sizes (Cohen’s d) are also given for differences between groups. Note that these estimates of effect size are based on univariate parametric tests, and therefore do not reflect the multivariate non-parametric analysis reported in the paper.

Measure	Mean consistent group (SD)	Mean inconsistent group (SD)	Cohen’s d
**ERRNI comprehension**	14.53 (1.92)	14.29 (2.02)	-0.13
**ERRNI MLU**	11.16 (1.99)	11.20 (2.21)	0.02
**Digit span**	18.33 (4.02)	17.23 (3.41)	-0.29
**WASI vocabulary**	62.03 (7.58)	61.39 (6.59)	-0.09
**YAA-R rapid naming**	1.88 (0.35)	1.76 (0.35)	-0.35
**TOWRE overall standard score**	100.05 (16.89)	93.94 (14.25)	-0.38
**NEPSY oromotor sequences**	62.30 (4.25)	62.13 (5.71)	-0.03
**NEPSY non-word repetition**	41.33 (4.10)	41.19 (3.49)	-0.03
**TOAL sentence assembly**	14.45 (3.39)	13.61 (3.16)	-0.25
**CCSR language**	9.21 (3.82)	8.45 (3.46)	-0.20
**CCSR pragmatics**	9.93 (3.25)	9.19 (4.19)	-0.21
**CCSR social**	10.71 (3.58)	9.84 (3.89)	-0.24

Supplementary Table 2: Means and standard deviations (SD) for control and developmental disorder (DD) groups on each of the 12 language measures. Effect sizes (Cohen's d) are also given for differences between groups. Note that these estimates of effect size are based on univariate parametric tests, and therefore do not reflect the multivariate non-parametric analysis reported in the paper.

Measure	Mean control group (SD)	Mean DD group (SD)	Cohen's d
**ERRNI comprehension**	14.65 (1.92)	14.36 (1.96)	-0.15
**ERRNI MLU**	11.87 (1.93)	10.79 (2.02)	-0.54
**Digit span**	19.76 (3.23)	17.03 (3.87)	-0.75
**WASI vocabulary**	62.38 (7.22)	61.54 (7.33)	-0.12
**YAA-R rapid naming**	2.04 (0.24)	1.74 (0.36)	-0.96
**TOWRE overall standard score**	108.19 (11.19)	92.73 (16.17)	-1.06
**NEPSY oromotor sequences**	63.41 (3.79)	61.61 (5.05)	-0.39
**NEPSY non-word repetition**	42.32 (3.52)	40.72 (4.03)	-0.42
**TOAL sentence assembly**	14.76 (2.85)	13.89 (3.56)	-0.26
**CCSR language**	10.22 (3.89)	8.29 (3.47)	-0.53
**CCSR pragmatics**	10.32 (3.02)	9.37 (3.79)	-0.27
**CCSR social**	11.49 (2.80)	9.88 (3.99)	-0.44
